# A Comprehensive Approach to Motorcycle-Related Head Injury Prevention: Experiences from the Field in Vietnam, Cambodia, and Uganda

**DOI:** 10.3390/ijerph14121486

**Published:** 2017-11-30

**Authors:** Greig Craft, Truong Van Bui, Mirjam Sidik, Danielle Moore, David J. Ederer, Erin M. Parker, Michael F. Ballesteros, David A. Sleet

**Affiliations:** 1 AIP Foundation, 12 B Ngoc Khanh Street, Ba Dinh District, Hanoi 100000, Vietnam; bvtruong1973@gmail.com (T.V.B.); mirjam.sidik@aipf-vietnam.org (M.S.); daniellemoore827@gmail.com (D.M.); 2 U.S. Centers for Disease Control and Prevention, 4770 Buford Highway, MS F-62, Atlanta, GA 30341 USA; davidederer@gatech.edu (D.J.E.); eparker@cdc.gov (E.M.P.); mballesteros@cdc.gov (M.F.B.); dds6@cdc.gov (D.A.S.)

**Keywords:** motorcycle, helmet, injury prevention, road safety

## Abstract

Motorcyclists account for 23% of global road traffic deaths and over half of fatalities in countries where motorcycles are the dominant means of transport. Wearing a helmet can reduce the risk of head injury by as much as 69% and death by 42%; however, both child and adult helmet use are low in many countries where motorcycles are a primary mode of transportation. In response to the need to increase helmet use by all drivers and their passengers, the Global Helmet Vaccine Initiative (GHVI) was established to increase helmet use in three countries where a substantial portion of road users are motorcyclists and where helmet use is low. The GHVI approach includes five strategies to increase helmet use: targeted programs, helmet access, public awareness, institutional policies, and monitoring and evaluation. The application of GHVI to Vietnam, Cambodia, and Uganda resulted in four key lessons learned. First, motorcyclists are more likely to wear helmets when helmet use is mandated and enforced. Second, programs targeted to at-risk motorcyclists, such as child passengers, combined with improved awareness among the broader population, can result in greater public support needed to encourage action by decision-makers. Third, for broad population-level change, using multiple strategies in tandem can be more effective than using a single strategy alone. Lastly, the successful expansion of GHVI into Cambodia and Uganda has been hindered by the lack of helmet accessibility and affordability, a core component contributing to its success in Vietnam. This paper will review the development of the GHVI five-pillar approach in Vietnam, subsequent efforts to implement the model in Cambodia and Uganda, and lessons learned from these applications to protect motorcycle drivers and their adult and child passengers from injury.

## 1. Introduction

Among the approximately 1.2 million lives lost to road trauma in the world each year, motorcyclists account for nearly one in four [1]. Head injuries are the leading cause of death in fatal motorcycle crashes [2]. Despite evidence that wearing a helmet can reduce the risk of head injury by 69% and death by 42% [3], helmets are widely underused, particularly in low and middle-income countries where motorcycles have rapidly become the primary means of transport. In countries where a substantial portion of road users are motorcyclists, increasing helmet use has the potential to substantially reduce road traffic injuries and deaths and the associated personal and societal costs.

To address the growing problem of injuries to motorcyclists in Vietnam and the low use of helmets, the Asia Injury Prevention Foundation (AIP Foundation) was established in Vietnam in 1999. Since that time, the AIP Foundation has designed programs and interventions to (1) improve public understanding of the safety value of helmet wearing; (2) provide access to high quality and low-cost helmets; and (3) evaluate the impact of policies that reinforce helmet use.

After years of development and implementation programs in Vietnam, AIP Foundation developed a five-pillar approach to increase helmet use in low- and middle-income countries where motorcycle-related head injury is prevalent. This approach was the foundation for the Global Helmet Vaccine Initiative (GHVI). GHVI was built on the premise that population-level changes in helmet use can reduce motorcycle-related head injury, but require a comprehensive approach. This paper describes the development and implementation of the GHVI five-pillar approach in Vietnam, its more recent application in Cambodia and Uganda, and lessons learned from these experiences.

### The Global Helmet Vaccine Initiative

The GHVI model includes five strategies, or pillars, to increase helmet use (Table 1). The strategies include (1) targeted programs to reach the highest risk populations (e.g., children and commercial taxi drivers); (2) helmet accessibility and affordability (building a helmet production factory and developing helmet standards, distributing helmets through retail outlets); (3) public awareness and education to help build public support; (4) support of institutional policies such as evidence-based policies for helmet use and standards for helmet safety; and (5) research, monitoring, and evaluation of efforts to improve helmet wearing. 

The AIP Foundation codified the GHVI approach into five pillars in 2009 and, with support from the FIA Foundation and the World Bank, scaled up GHVI activities in 2010 to Cambodia and Uganda, with a goal of putting a helmet on every head in support of the Decade of Action for Road Safety (2011–2020) [4]. The National Center for Injury Prevention and Control and the Center for Global Health at the U.S. Centers for Disease Control and Prevention provided technical support for monitoring and evaluation related to the GHVI activities.

The GHVI approach was implemented in three countries: Vietnam, Cambodia, and Uganda. These countries had high levels of motorcycle use, low helmet wearing rates, and significant burdens of death and injury resulting from motorcycle-related head injury [5]. In each country where GHVI was implemented, the pillars were the foundation for interventions and actions, but were tailored to each country’s unique setting and circumstances.

## 2. Vietnam

### 2.1. Background

In the 1990s, the number of motorized vehicles on Vietnam’s roads quadrupled [6]. The motorcycle emerged as the primary mode of transport in Vietnam and was often used as the family vehicle, carrying one child or more. Annual road traffic fatalities and injuries increased by more than 250% [6]. Despite attempts by the government to legislate and enforce motorcycle helmet use in the early 2000s, helmet use rates remained low as of 2005 [7]. Motorcycle helmet laws were only enforceable on select national and provincial roads [8]. Meanwhile, road traffic crash injuries continued to increase, costing Vietnam ~2.9% of their national gross domestic product [5].

### 2.2. Program Implementation

The AIP Foundation launched its first targeted program, *Helmets for Kids (HFK)*, in 2000 to improve helmet wearing among Vietnamese primary school children, grades 1–5. *HFK* provides free helmets to students, accompanied by road safety education at schools located near dangerous roads (http://aip-foundation.org/our-work/targeted-programs/helmet-safety/). The majority of students commute to and from school by motorcycle or bicycle, with few or none wearing helmets (Greig Craft, personal communications). In 2001, Vietnam adopted a child-specific helmet standard (TCVN 6979/2001), requiring all motorcycle helmets for children under the age of 16 years sold in Vietnam to be tested and certified for compliance with a set of quality criteria [9]. Since its inception, *HFK* has succeeded in increasing the rate of helmet use among children commuting to and from school via bicycle or motorcycle in target schools. In the 2013–2014 school year, the AIP Foundation’s internal program evaluation indicated that helmet wearing at the 38 *HFK* target schools in Vietnam increased from 24% at the beginning of the school year to 96% at the end of the school year (unpublished data, AIP Foundation).

When the AIP Foundation began working in Vietnam, high-quality, affordable, climate-appropriate helmets were scarce, indicating the need to increase helmet accessibility at a low cost. The absence of helmets designed for hot weather was identified as a major barrier to use for many people in Vietnam. The AIP Foundation built the PROTEC helmet factory in Hanoi in 2001. The PROTEC factory produces, tests, and distributes helmets. Profits from helmet sales are reinvested in AIP Foundation campaigns and used to subsidize free and discounted helmets.

Despite these successes, helmet use in the population remained low—less than 30% use on average, and 10% on city roads, according to observational surveys in 2006 [7]. Where helmet use was compulsory, helmet use rates were higher, according to studies in three major areas of Vietnam [7]. Combining public awareness, helmet education, and institutional policy change, the AIP Foundation began a national mass media campaign and met with policy-makers to encourage a mandatory helmet law that would apply to all roads and to all motorcyclists. The campaign addressed widespread barriers to helmet use, such as appearance, discomfort, and distance traveled, and reasons why such barriers are minor by comparison to the lifetime damage and cost associated with non-helmet use. Traffic police were trained in enforcement in preparation for a major shift in policy favoring a mandatory helmet law in Vietnam. On 29 June 2007, a helmet law (Resolution 32) was enacted by the legislature, and almost immediately helmet use increased to over 90% [8,10]. Motorcycle-related fatalities decreased by 46% between 2005 and 2012 [6], demonstrating the essential role played by education, political will, and enforcement in increasing helmet use. The helmet use rate has since dropped slightly, but remains above 90% [5].

Continuous research, monitoring, and evaluation efforts were implemented to determine the short- and long-term effectiveness of the program. A baseline study conducted by the AIP Foundation on motorcyclists’ helmet-related knowledge, attitude, and reported behavior identified further barriers to wearing helmets, which helped shape subsequent campaign messages. Over the nine years following the campaign and enforcement of the law, millions of dollars in medical costs were saved. A recent analysis indicates that nearly $50 million in acute care and lost income alone were averted in the first year after the law was enacted [11]. This evidence, together with the effectiveness of setting and enforcing the helmet law, provided the rationale for expanding the five-pillar approach to Cambodia and Uganda.

### 2.3. Implementation Challenges

Vietnam’s 2007 helmet law applied to all riders on all roads. However, without clear legal stipulations for child helmet use—children under the age of 14 years could not be given fines—compliance among this group did not correspond with adult use [12]. A subsequent passage of Decree 34 in May 2010 required children aged 6 years and older to wear helmets on motorcycles, and adults carrying un-helmeted children were now also subject to fines. However, child helmet wearing rates remained low [13]. In 2015, the government’s National Child Helmet Action Plan (NCHAP) addressed this issue through a multi-pronged approach, including a public awareness campaign, school-based interventions, and enhanced police enforcement; this resulted in an increase in child helmet use from 36 to 57% in Vietnam’s three major cities, Hanoi, Ho Chi Minh City, and Danang [14]. Another remaining challenge is the prevalence of sub-standard helmets [15]. Widespread availability of cheap non-standard helmets on the market and lack of government coordination to enforce the standard are some contributing factors.

## 3. Cambodia 

### 3.1. Background 

From 2007 to 2013, road traffic deaths in Cambodia increased by 50%, while registered vehicles—of which more than 80% are motorized two- or three-wheelers—skyrocketed from less than 200 thousand to nearly 2.5 million [5,16]. Between 2007 and 2012, only about 10% of fatally injured motorcyclists were wearing helmets at the time of the crash, and 76% of motorcyclist deaths involved head injuries [17]. Until a recent law with enforcement beginning in January 2016, helmets in Cambodia were compulsory for motorcycle drivers only—not passengers. Consequently, helmet use among drivers was substantially higher than that among passengers, 68% vs. 8% in November 2012 [18].

### 3.2. Program Implementation

The AIP Foundation established GHVI in Cambodia in 2010 to increase motorcycle driver and passenger helmet use, including child passengers. Prior to 2010, the AIP Foundation first introduced the *Helmets for Kids (HFK)* targeted program in Cambodia in 2006. Based on findings from AIP Foundation surveys in 2010–2011 that helmets were misconceived as unnecessary for young children and that schools were important sources of traffic safety information for children, the *HFK* program was refined and expanded under GHVI [19]. From 2011 to 2013, *HFK* provided free helmets and road safety education to 6853 students at nine schools. Teachers were engaged through trainings and workshops. School policies encouraged helmet wearing as part of the students’ uniform. Following program implementation, helmet use among students traveling to and from intervention schools increased from 0 to 88%, on average, by the end of each school year [20]. Helmet use remained less than 1% in control schools.

GHVI implemented survey research in Cambodia to inform public awareness and education campaigns to increase passenger helmet use. Baseline survey data indicated that driving destination, forgetting, and inconvenience were the primary reasons that motorcycle passengers reported not wearing helmets. Messages promoting passenger helmet use were disseminated on TV, radio, billboards, and digital media. The campaign resulted in a high recall of key messages and intended behavior change. However, passenger helmet use, including among children, remained low [21].

GHVI encouraged institutional policy change by generating public support for the passage and enforcement of a passenger helmet law. In October 2011, GHVI called for stakeholders to co-sign a letter requesting the inclusion of children in the draft passenger helmet law, to which the government consented. In January 2013, GHVI submitted a joint statement endorsed by over 100 stakeholders and 4700 Cambodian citizens to the Minister of Transport and Chairman of the National Road Safety Committee calling for the swift enactment of the passenger helmet law. The rationale for these actions was entirely supported by research, monitoring, and evaluation survey data, which identified high levels of intended behavior change and broad public support for the mandate.

GHVI also projected the potential life- and cost-savings of passing a passenger helmet law, and presented the results to policy-makers. Framing the problem and potential benefits of the solution in this manner was critical in discussions with the government. In August 2014, three months after presenting these calculations, decision-makers approved a new law requiring passenger helmet use. In December 2014, the National Assembly passed the law, which was officially promulgated in January 2015, and has been enforced since January 2016, paving the way for drivers and passengers to be protected by wearing helmet while driving or riding a motorcycle in Cambodia. Notably, while the law mandates helmet use for passengers, including children aged 3 years and older, the associated sub-decree only includes fines for those aged 15 years and older. 

### 3.3. Implementation Challenges

Despite the passage of the helmet law, there were no controls for the type or quality of the helmets worn. Each year Cambodia imports from other countries a large number of low-quality helmets with minimal or no safety testing. The Cambodian government and stakeholders agree on the need for improved access to high-quality helmets that meet safety standards. To improve high-quality helmet accessibility, GHVI has encouraged the government to establish a helmet testing laboratory to ensure that all Cambodians have access to safe and affordable helmets made locally or imported from abroad.

## 4. Uganda

### 4.1. Background

Between 2007 and 2013, the burden of motorcycle deaths in Uganda increased dramatically, with deaths among riders on motorized two- or three-wheelers increasing from 7 to 30% [5,16]. A comprehensive motorcycle helmet mandate in Uganda’s 2004 Traffic and Road Safety Act requires that motorcycle drivers and passengers wear helmets, but enforcement has been low [5]. Consequently, helmet use in 2013 was estimated to be 46% for drivers and 1% for passengers [5].

Compared with Cambodia and Vietnam, vehicle ownership in Uganda is low [5]. Commercial motorcycle taxi operators, commonly referred to as boda bodas, have become a ubiquitous and inexpensive, but dangerous, means of transport [22,23]. Only 31% of boda boda drivers wore helmets in 2011, and boda boda crashes account for 41% of all trauma patients at the main referral hospital in Kampala [24,25].

### 4.2. Program Implementation

The AIP Foundation established GHVI in Uganda in 2010 as a means to increase helmet use among motorcyclists, specifically commercial taxi drivers, and to boost enforcement of the law. Boda bodas have a reputation for risky behavior, leading to the perception that they are largely at fault for crashes in Kampala [23]. GHVI targeted boda bodas, because they are:

- At high risk;
- Incentivized to reduce crashes and injuries to themselves and their passengers as a matter of sound business practice;
- Well-organized and easy-to-reach through a Boda Boda Association; and
- Highly visible, giving them the potential to be change agents, further increasing the impact of the initiative through peer-to-peer influence.

From 2011 to 2015, GHVI conducted 38 workshops for approximately 3800 boda boda drivers in Kampala, providing helmet safety education and free helmets. At the conclusion of the workshops, each was given a PROTEC helmet, manufactured in Vietnam and tested against the Uganda safety standard. A public awareness and education strategy was also developed for boda boda drivers. A radio campaign featuring 30-s public service announcements and radio talk formats was aired on radio stations in Kampala, as radio is a frequently relied on media among boda boda drivers who spend time listening to the radio between rides. The message content for radio public service announcements was based on formative survey research indicating that “comfort” and “expense” were key barriers to helmet use. Based on the AIP Foundation’s helmet observations, helmet use increased from 31 to 49% among boda boda drivers in Kampala between 2011 and 2013 (unpublished data, AIP Foundation). In the control city, Mbale, where no activities were conducted, helmet use increased from 12 to 20%. Following subsequent efforts, including additional traffic and helmet safety workshops, communication, and enforcement, helmet use among boda boda drivers increased to 77% in the target city of Kampala in 2015 [26].

To address lack of enforcement, GHVI worked to increase understanding of the importance of helmet use among boda boda drivers and to support the comprehensive helmet mandate. GHVI rallied support from more than 30 stakeholders, including the traffic police, the Boda Boda Association, and local experts for sustaining and improving enforcement actions, including developing a Ugandan helmet standard. Stakeholder involvement in helmet safety has gathered momentum in a variety of ways.

In February 2012, the government passed a helmet standard, based on the Vietnam standard (QCVN2/2008/BKHCN) and the GHVI-developed “MCH1 Specifications for head protection for motorcyclists” [27], intended to provide a technically feasible standard that can be implemented in locations that currently do not have an existing motorcycle helmet standard and do not possess the technical expertise to develop their own motorcycle helmet test procedures. The standard incorporates affordable and climate-appropriate helmets and better equips police officers to identify substandard helmets.

In May 2013, the Boda Boda Association and Makerere University partnered with GHVI to present to Parliament on findings of the burden of head injuries to boda boda drivers in a crash, as well as the potential impact of widespread helmet use on Kampala’s health system. In September 2013, the Kampala Capital City Authority began registering boda bodas. Prior to this action, there was no registration and no requirements to become a boda boda driver. GHVI invited the first 400 registrants from each district to participate in a helmet safety workshop and receive a free helmet.

### 4.3. Implementation Challenges

Despite clear rationale for the health and safety benefits for helmet use and a mandate to wear helmets and strong enforcement, motorcyclists will not use helmets if they are not available and affordable. High-quality, affordable helmets are not widely accessible in Sub-Saharan Africa, and the best helmets are normally quite expensive. Although there was a lack of regional helmet accessibility, GHVI was able to provide helmets to boda bodas from its helmet factory in Vietnam. Moreover, poor data quality has made it challenging to collect surveillance data and routine morbidity and mortality data from local hospitals to monitor changes in motorcycle injuries and fatalities.

## 5. Lessons Learned

The application of GHVI to Vietnam, Cambodia, and Uganda has resulted in four key lessons from our experience of using a comprehensive approach to promote the use of helmets. First, motorcycle drivers and passengers, including children, are more likely to wear helmets when helmet use is mandated and enforced. For example, Vietnam saw a substantial increase in helmet use after their legislature enacted and enforced a law.

Secondly, programs targeted to at-risk motorcyclists, combined with improved awareness among the broader population, can produce the evidence and public support needed to leverage action by key decision-makers. For example, public awareness, education, and evidence-based policy strategies led to the approval and enforcement of a passenger helmet law in Cambodia. Similarly, helmet use did not rise substantially in Vietnam until targeted programs and public awareness education improved the understanding of helmet safety and the importance of helmets to prevent head injury.

Third, population-level change is most likely to occur when comprehensive strategies are used in tandem. Single strategies on their own may have limited effectiveness. For example, even with a law mandating helmet use, the public must still know about the law and be willing to comply with it. Awareness and education can help overcome some of these challenges. In Uganda, for example, passing a law mandating helmet use alone seemed to have limited impact on helmet use. Employing other strategies simultaneously, such as boda boda education, awareness of the benefits of helmet use, and the provision of a free helmet, seemed to increase acceptance, use, and compliance with the law among boda boda drivers.

Finally, poor helmet accessibility remains a challenge to the successful expansion of GHVI’s approach in Cambodia and Uganda, as compared to Vietnam. While helmet use increased in target groups when helmets were provided, to achieve population-level improvement, safe helmets must be made widely available and affordable. In Vietnam, for example, a helmet factory producing quality helmets at a fair price helped more people find, buy, and use helmets. It also decreased reliance on imported helmets from abroad, many of which are unsafe and/or expensive.

## 6. The Way Forward

The AIP Foundation is using these experiences to help inform extending the GHVI approach to other countries, such as Thailand, which have similarly low helmet use rates. In Vietnam, the AIP Foundation is continuing to work to improve child helmet use, which remains dangerously low, despite high adult use, even with the 2010 law mandating helmet use for children aged 6 years and older. In Cambodia, the AIP Foundation and its partners will continue to build capacity with local and provincial traffic police to enforce the recent passenger helmet law. In Uganda, the AIP Foundation and its partners will continue to implement the GHVI approach based on lessons learned that from this report. Similar efforts toward expansion are underway in Tanzania. Ideally, others in health and traffic safety can use the lessons learned here to test and adapt the AIP Foundation’s five-pillar approach in other contexts. This expansion may help achieve the AIP Foundation’s goal “to put a helmet on every head” in support of the Decade of Action for Road Safety (2011–2020), and to make progress toward the 2030 UN Sustainable Development Goals, Target 3.6, to halve the number of global deaths and injuries from road traffic crashes by 2020 [28]

## 7. Conclusions

The Global Helmet Vaccine Initiative (GHVI) was established to increase helmet use in countries, such as Vietnam, where a substantial portion of road users are motorcyclists and where helmet use is low. The GHVI five-pillar approach consists of targeted programs, helmet access, public awareness, institutional policies, and monitoring and evaluation. This approach was successfullyused in Vietnam and implementation of the model was adapted and implemented in Cambodia and Uganda. Adaptation and expansion of this model in other countries may help support global goals and targets to reduce road traffic injuries, worldwide.

## Figures and Tables

**Table 1:** Five pillars of the Global Helmet Vaccine Initiative.

Pillars	Description
1. Targeted programs	Promoting helmet use through programs targeted at high-risk populations, such as children and commercial taxi drivers; delivering tailored educational messages on helmets’ safety value; building support among peers and institutions of the target population.
2. Helmet accessibility and affordability	Providing access to high-quality, low-cost, climate-appropriate helmets; testing helmets to meet helmet safety standards; distributing helmets through retail outlets.
3. Public awareness and education	Improving public knowledge of helmets’ safety value; reinforcing the benefits of helmet use, consequences of non-use, and the returns on investment from reduced costs to society.
4. Institutional Policies	Supporting evidence-based policies for helmet use and standards for helmet safety.
5. Research, monitoring, and evaluation	Continuously measuring the results to monitor progress toward increasing helmet use, public attitudes toward use, healthcare costs and savings from helmet use, and burden to society from non-use; disseminating promising results and outcomes to the broader public and decision-makers.
